# Aggregated and disaggregated data about default emission factors in emissions accounting methods from the waste sector

**DOI:** 10.1016/j.dib.2018.09.094

**Published:** 2018-10-04

**Authors:** Amani Maalouf, Mutasem El-Fadel

**Affiliations:** Department of Civil & Environmental Engineering, American University of Beirut, Lebanon

## Abstract

The dataset presented in this article is related to the research article entitled “Towards improving emissions accounting methods in waste management: A proposed framework” (Maalouf and El-Fadel, 2019) [1] that examines the variability in aggregated and disaggregated emissions from waste management when using commonly adopted international methods (the UN IPCC 2006 Guidelines, the US EPA WARM, the EU EpE protocols, the Canadian IWM, and the UK IWM-2). The dataset presents the aggregated and disaggregated emission factors (EFs) used in existing accounting methods to estimate emissions from the waste sector. The EFs were retrieved from accounting methods to clarify their contribution to variability in estimating emissions across methods. The data contains three parts: aggregated EFs per tonne of waste category for individual waste management processes; disaggregated EFs per management process for a tonne of waste type; and emission flow diagrams of waste management systems for tested methods.

**Specifications table**

**Table:** 

Subject area	*Environmental engineering*
More specific subject area	*Emission accounting from waste management*
Type of data	*Tables, figures, and text*
How data was acquired	*Secondary data sources (e.g. reports, literature, and existing models/software)*
Data format	*Raw and analyzed data*
Data source location	*Department of Civil & Environmental Engineering, American University of Beirut, Lebanon*
Data accessibility	*Data is included in this article*
Related research article	*Maalouf, A., El-Fadel, M. Towards improving emissions accounting methods in waste management: A proposed framework. J. Clean. Prod. 206 (2019) 197-210.* doi: 10.1016/j.jclepro.2018.09.014.

**Value of the data**

• The data consist of aggregated and disaggregated emission factors that are adopted in existing accounting methods to estimate emissions from the waste sector.
• A significant difference is evident in emission factors across tested methods.
• Data analysis accentuates the need for uniformity in emissions accounting methods and corresponding default parameters particularly emission factors.
• The data can guide the estimation process of emissions from the waste sector.
• The data can influence decision making when assessing emissions mitigation measures and reporting targets under the United Nations Framework Convention on Climate Change (UNFCCC) agreements or influence reduction targets using carbon credits to meet nationally determined contributions (NDCs) under the Paris Agreement.

## 1 Data

The data presented in this article provides details about emission factors (EFs) used in estimating emissions from the waste sector. The data clarifies the contribution to the variability in emissions when using commonly adopted international methods (the UN IPCC 2006 Guidelines [2], the US EPA WARM [3], the EU EpE protocols [4], the Canadian IWM [5], and the UK IWM-2 [6]. These methods were selected because they are publically accessible, widely reported in the literature, and adopted by cities or countries where they were originally developed [7], [8], [9], [10]. The Intergovernmental Panel on Climate Change (IPCC) guidelines in particular were supposedly put forth to standardize between methods at a global scale. The data consist of disaggregated EFs expressed in metric tonnes of CO_2_ equivalents (MTCO_2_E) per characteristic unit and refer to EFs separated by waste category, gas, waste processes, and type of emissions (direct or indirect). It also includes details on aggregated EFs (MTCO_2_E/ tonne of waste), which are the combined outcome of indirect-upstream, direct-operational, and indirect-downstream emissions from treating one tonne of waste by individual waste management processes. Note that waste always refers to wet waste. Moreover, given that the 100-year global warming potential (GWP_100_) for greenhouse gases (GHGs) has evolved with time as outlined in (Table 1), the GWP_100_ was adjusted in all methods to follow the IPCC, 1995 [11] reference definition. The latter was selected as a reference in all methods because most of them rely on the IPCC (1995) by default. Note that changing the GWP_100_ affect emissions estimation. For instance, WARM uses IPCC, 2007 [12] resulting in 19% increase in GWP_100_ of CH_4_, in comparison to IWM-2 that uses IPCC, 1995 [11].

**Table 1:** GWP for 100-year time horizon.

**GHGs**	**Symbol**	**First assessment report (FAR) IPCC**[13]	**Second assessment report (SAR) IPCC**[11]	**Third assessment report (TAR) IPCC**[14]	**Fourth assessment report (AR4) IPCC**[12]	**Fifth assessment report (AR5) IPCC**^a^[15]
Carbon dioxide	CO_2_	1	1	1	1	1
Methane	CH_4_	21	21	23	25	34
Nitrous oxide	N_2_O	290	310	296	298	298

a Including climate-carbon feedbacks.

**Fig. 1:**
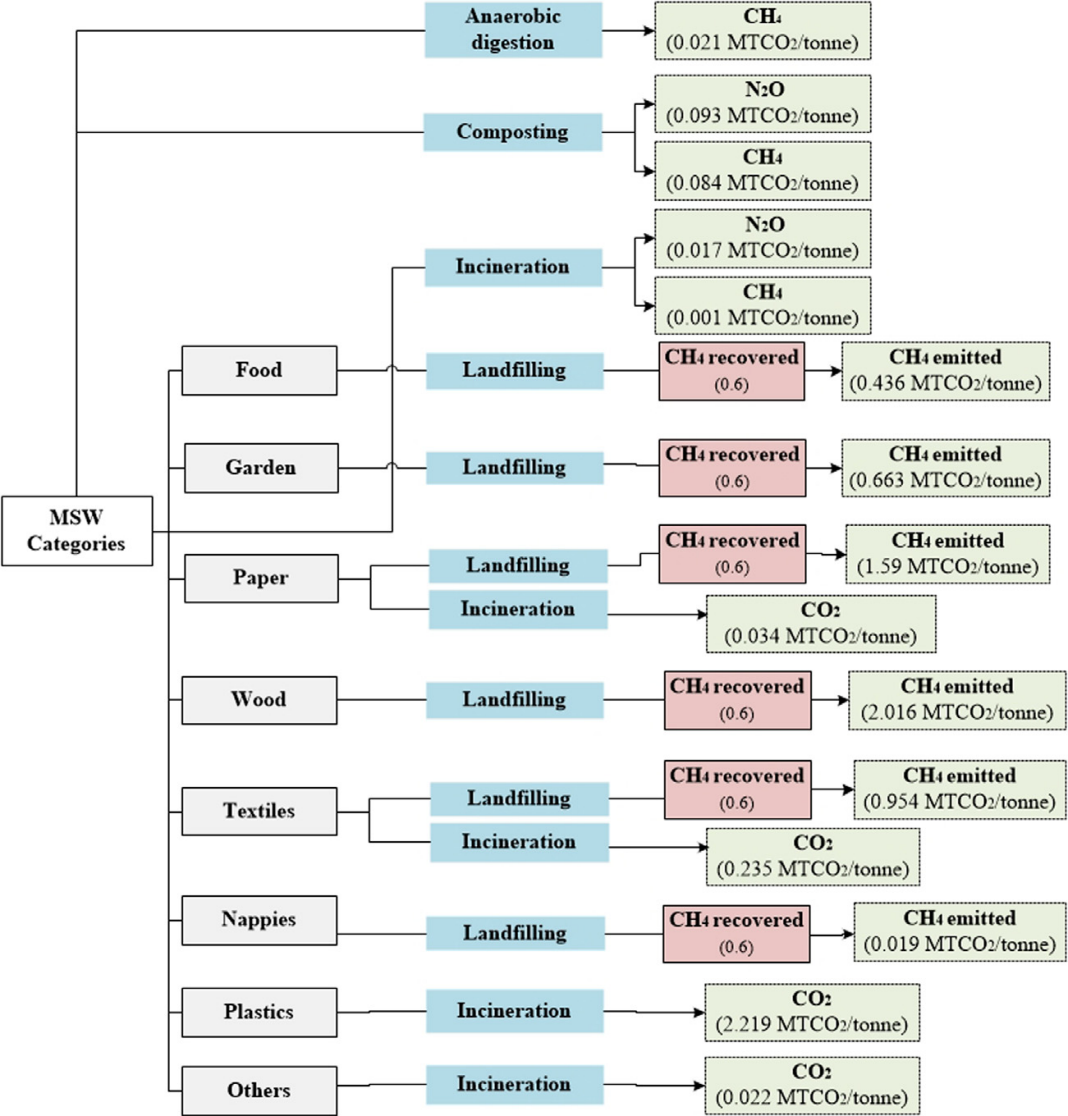
IPCC-2006.

**Fig. 2:**
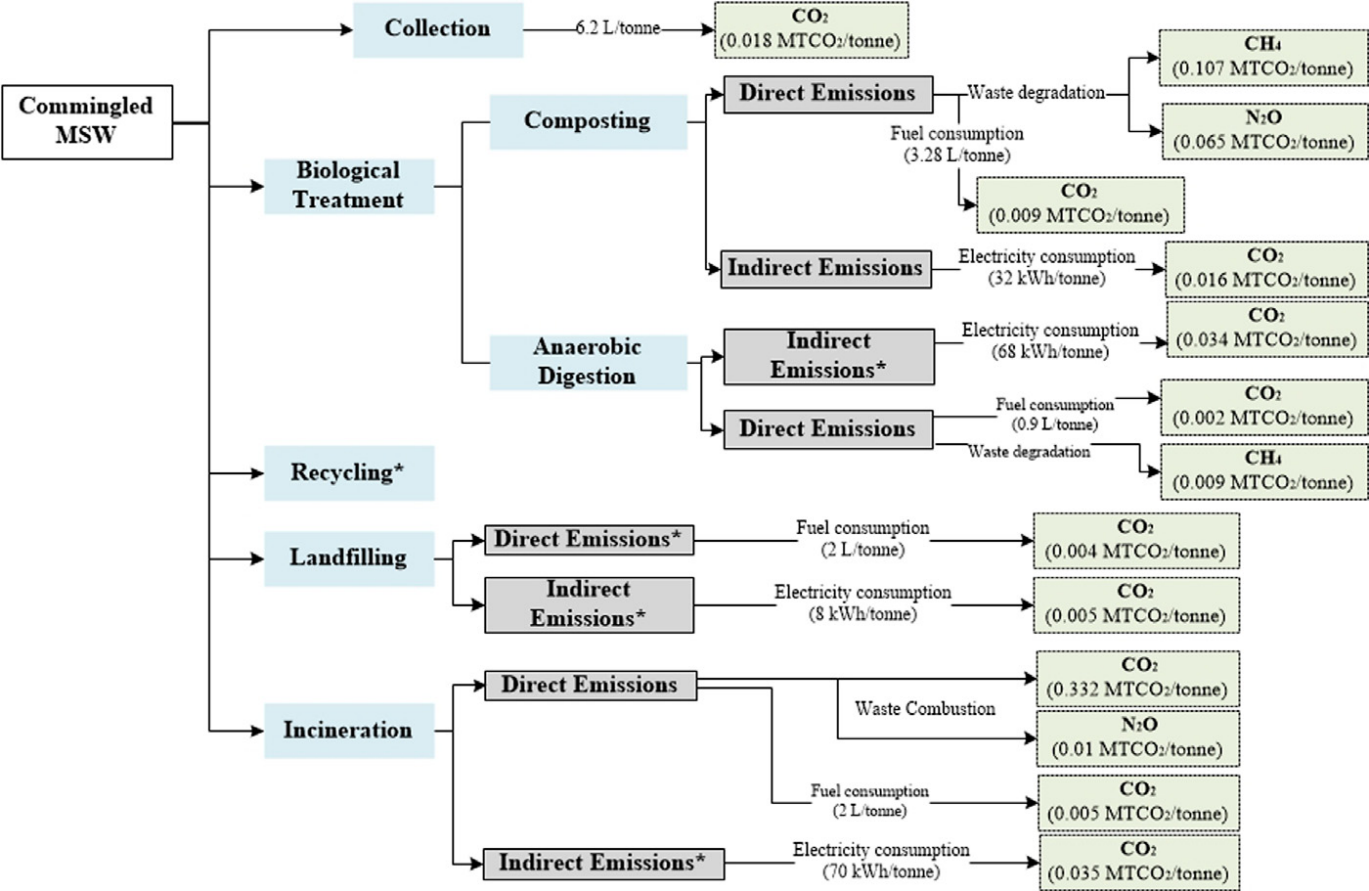
EpE protocol. *Note that EpE does not provide methodologies to estimate avoided emissions from recycling, energy recovery from anaerobic digestion, landfill, and incineration as well as direct emissions from waste degradation during landfilling.

**Fig. 3:**
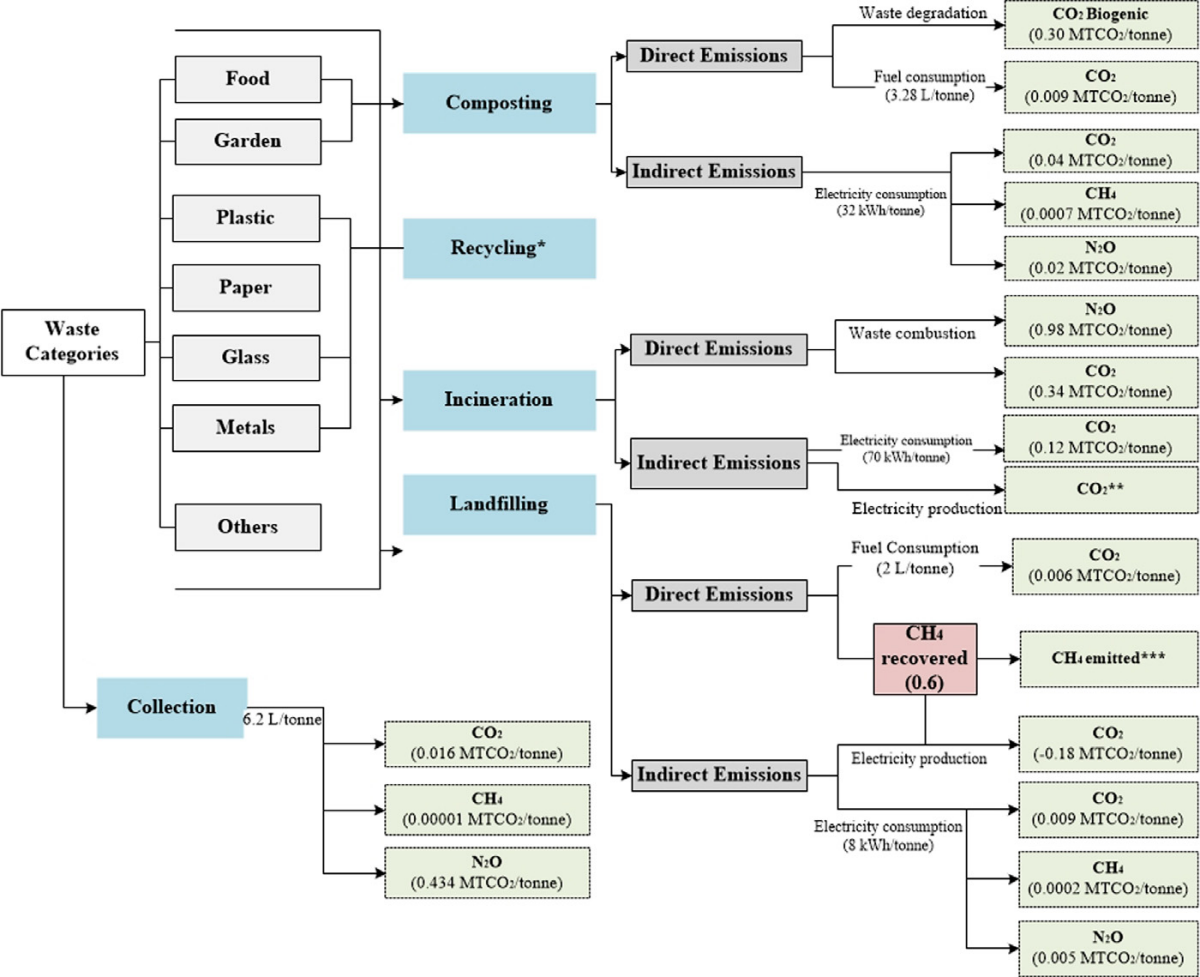
IWM. *During recycling IWM considers avoided emissions from plastics, glass, and metals **During incineration IWM only considers CO_2_ emissions from paper, glass, metals, plastics, food, and others. ***During landfilling IWM only considers CH_4_ emissions from paper, and food.

**Fig. 4:**
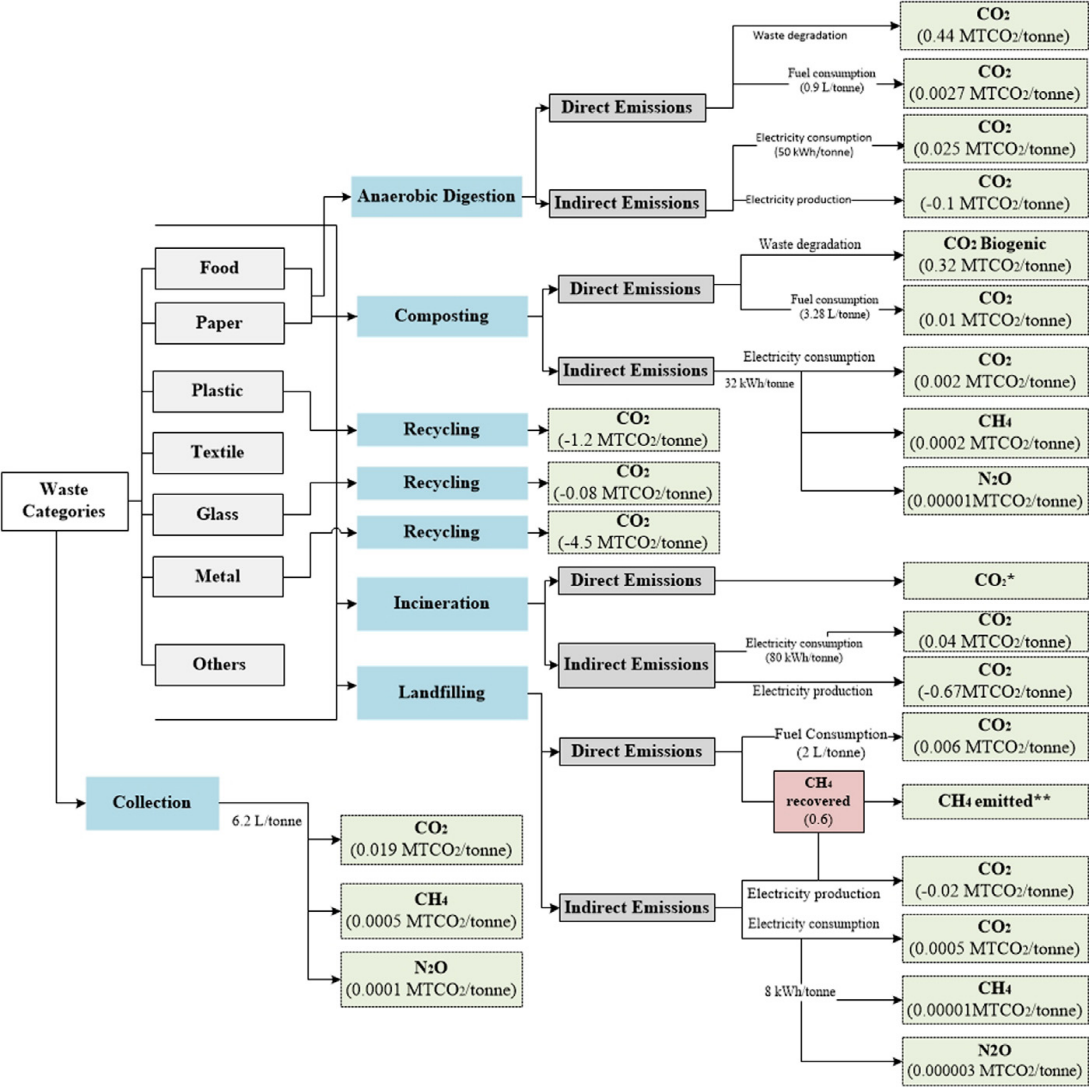
IWM-2. *During incineration IWM-2 only considers CO_2_ emissions from paper, glass, plastics, textiles, food, and others **During landfilling IWM-2 only considers CH_4_ emissions from paper, textiles, and organics.

**Fig. 5:**
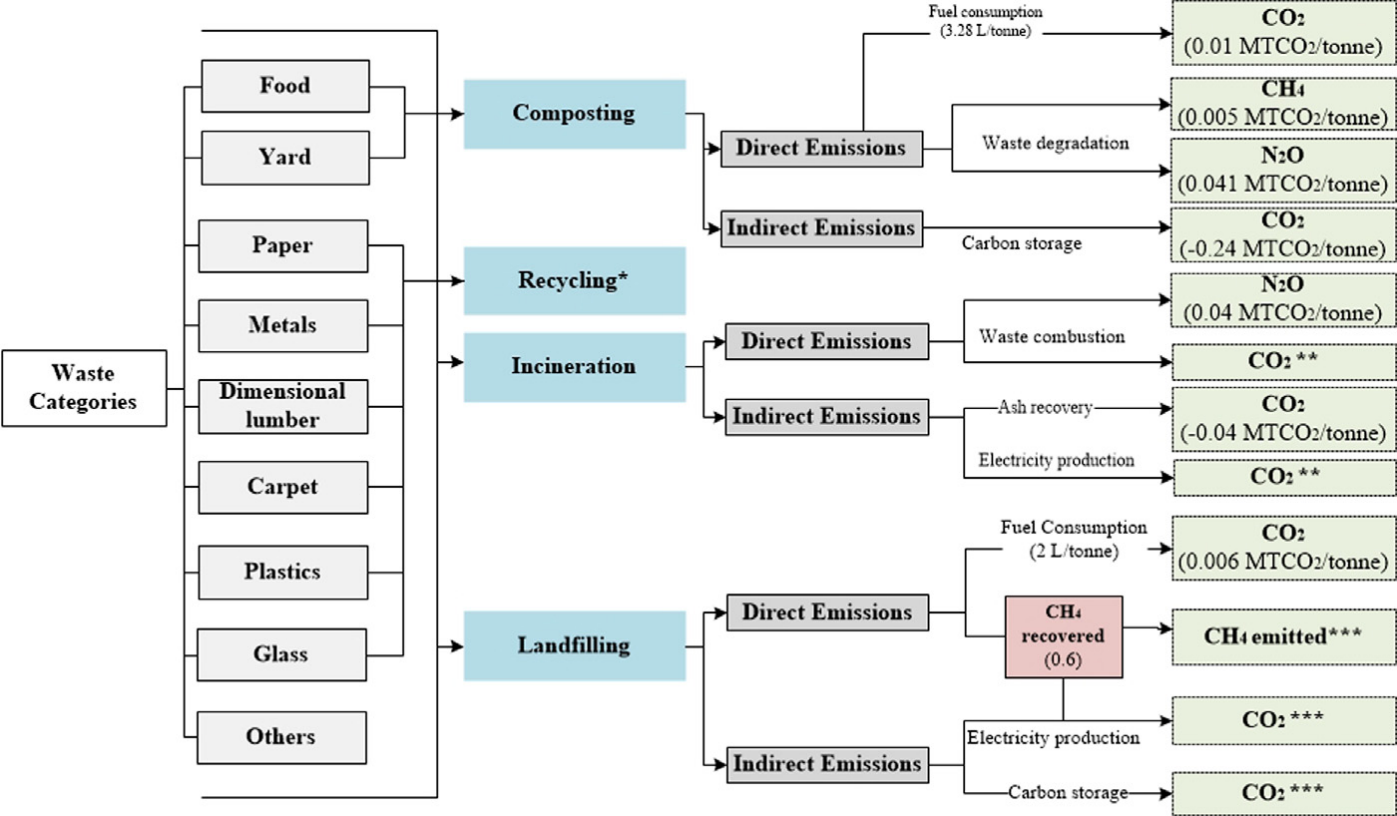
WARM. *During recycling WARM considers avoided emissions from paper, plastics, glass, carpet, dimensional lumber, and metals **During incineration WARM only considers CO_2_ emissions from paper, plastics, textiles, wood, food, and others ***During landfilling WARM only considers CH_4_ emissions from paper, food, wood, and others.

Table 2, Table 3, Table 4, Table 5, Table 6 show the aggregated default EFs per tonne of waste category for individual waste management processes. A further illustration of the EFs (disaggregated and aggregated) adopted in each method is presented in Table SM1 (in the Supplementary Material). Flow diagrams of waste management systems with energy sources and resulting emissions for each method are displayed in Fig. 1, Fig. 2, Fig. 3, Fig. 4, Fig. 5.

**Table 2:** Emission factors related to waste collection.

**Method**	**Type of EF**	**Values**	**Variability in EFs(%)**^d^
IPCC-2006 a	Not considered		
EpE	Aggregated b	0.018	11–289
	Disaggregated c	EF_fuel CO2_= 0.0026	
IWM	Aggregated	0.07	70–74
	Disaggregated	EF_fuel CO2_ = 2.6 × 10^-3^	
		EF_fuel CH4_ = 2.8 × 10^-6^	
		EF_fuel N2O_ = 0.007	
IWM-2	Aggregated	0.021	14–233
	Disaggregated	EF_fuel CO2_ = 0.003	
		EF_fuel CH4_ = 7.7 × 10^-5^	
		EF_fuel N2O_ = 2.2 × 10^-6^	
WARM	Aggregated	0.02	10–250
	Disaggregated	EF_fuel CO2_ = 0.003	

a The IPCC does not account for emissions from collection of waste within the waste sector Such emissions are embedded within the Transport sector under Energy.

b Aggregated Emission Factor (EF): (MTCO_2_E per tonne of waste category) (GWP_100_; IPCC [11]).

c Disaggregated EF_fuel g_ = Emission factor of gas *g* from fuel combustion (MTCO_2_E/Liters of fuel) with 6.2 L of fuel consumed/tonne of waste collected in the study area GWP_100_; IPCC [11].

d The absolute variability in EFs is calculated with respect to each method.

**Table 3:** Aggregated emission factors per tonne of waste category recycled (MTCO_2_E per tonne of waste category).

**Waste Category**	**IWM**	**IWM-2**	**WARM**
Paper	−0.83		−3.52
Plastics	−4.53	−1.20	−0.98
Textiles		−5.87	−2.37
Wood			−2.46
Glass	−0.92	−0.09	−0.28
Metals	−1.99	−4.55	−3.97

**Table 4:** Aggregated emission factors per tonne of waste category composted (MTCO_2_E per tonne of waste category).

**Waste Category**	**IPCC-2006**	**EpE**	**IWM**	**IWM-2**	**WARM**
Food			0.066	0.012	−0.184
Garden					−0.155
Other	0.177^a^	0.175^b^			

a Considers total mass of municipal solid waste (MSW) treated.

b Considers CH_4_ emissions from the Organic fraction of MSW and N_2_O emissions from MSW.

**Table 5:** Aggregated emission factors per tonne of waste category landfilled (MTCO_2_E per tonne of waste category).

	**IPCC-2006**^a^	**EpE**^b^	**IWM**	**IWM-2**	**WARM**
Food	0.436		0.496	0.832	0.578
Paper	1.590		0.684	0.832	0.036
Plastics	0				0.006
Textiles	0.954			0.832	0.006
Garden	0.663				0.988
Wood	2.016				-0.614
Glass					0.006
Metals					0.006
Other		0.009			1.242

a Emissions from landfilling are calculated based on regulatory methodologies recommended by local authorities. It also considers direct emissions (from permanent thermal facilities and on-site mobile equipment) and indirect emissions (from electricity or heat consumption), and avoided emissions (from electricity and heat recovery).

b LCA-based methods consider methane emissions from landfilling of waste disposed in a selected inventory year (using the gas yield method), over a 100-year time horizon, while other methods such as the IPCC-2006 [2] adopt the first order decay (FOD) that considers the cumulative emissions of waste deposited in previous years. Instead of accounting for emissions over a time-period and considering the accumulation of emissions for every year from previous years, year 0 was selected as the inventory year to account for the waste behavior of this year over a 100-year prediction.

**Table 6:** Aggregated emission factors per tonne of waste category incinerated (MTCO_2_E per tonne of waste category).

**Waste Category**	**IPCC-2006**	**EpE**	**IWM**	**IWM-2**	**WARM**
Food			−0.04	0.57	−0.12
Paper	0.03		−1.1	1.24	−0.42
Plastics	2.22		−1.71	2.65	1.56
Textiles	0.25			1.24	1.23
Garden					−0.19
Wood					−0.43
Glass			0.38	0.09	−0.02
Metals			0.5		−0.02
Other	0.022	0.382	−0.58	1.24	−0.01

## 2 Experimental design, materials, and methods

Data on EFs for various waste management processes was collected through secondary sources of accessible reports, literature, Guidelines, and models/software. The data was categorized into:

(1) Disaggregated EFs, which are by definition factors determined from a number of processes representing characteristics calculated per unit of activity; thus, they are expressed in MTCO_2_E per characteristic unit (tonne of municipal solid waste treated; kW h of electricity; Liter of Diesel fuel) using a GWP_100_, IPCC, 1995 [11]. EFs are fixed default values within every method except for the EpE method where the user can select EFs of recycling (adapted from USEPA/ICF, 2012 [3]) and landfilling (adapted from IPCC-2006 Guidelines [2]).
(2) Aggregated EFs is the combined outcome of disaggregated EFs expressed in MTCO_2_E per tonne of waste category. Note that LFG (landfill gas collected) = 0.6; Electricity consumed = 32 kW h/tonne of waste composted, 70–80 kW h/tonne of waste incinerated, 68–50 kW h/tonne of waste anaerobically digested, and 8 kW h/tonne of waste landfilled; Fuel consumed = ~2 l/tonne of waste landfilled, ~3.28 l/tonne of waste composted, and 0.89 l/tonne of waste anaerobically digested.
