# Supplementary material for: Aggregated and disaggregated data about default emission factors in emissions accounting methods from the waste sector
Source: Data Brief. 2018 Oct 4;21:568–75. doi: 10.1016/j.dib.2018.09.094 (PMC6199779; doi:10.1016/j.dib.2018.09.094)
Supplement: Supplementary file 2 — Supplementary material [file mmc2.docx]

# Supplementary Material

Table SM1. Aggregated and disaggregated emission factors per management process and tonne of waste type

| **Method ^(a)^** | **Type of EFs** | **Recycling** | **Composting** | **Anaerobic Digestion** | **Incineration** | **Landfilling** |
| --- | --- | --- | --- | --- | --- | --- |
| *By Waste Type* | | | | | | |
| *Food* |  | | | | | |
| IPCC-2006 | Aggregated  Disaggregated |  |  |  |  | 0.436  EF_Lf F CH4_ =1.09 |
| EpE |  |  |  |  |  |  |
| IWM | Aggregated  Disaggregated |  | 0.066  EF_fuel CO2_=0.0027  EF_elec CO2_=1.1x10^-3^  EF_elec CH4_=0.02x10^-3^  EF_elec N2O_ =0.6x10^-3^ |  | -0.04  EF_D I N2O_=0.33  EF_ID I_ = -0.37 | 0.496  EF_Lf F CH4_ =1.21  EF_Lf F N2O_=0.013  EF_fuel CO2_=0.0027  EF_elec CO2_=1.14x10^-3^  EF_elec CH4_ =0.02x10^-3^  EF_elec N2O_= 0.6x10^-3^ |
| IWM-2 | Aggregated  Disaggregated |  | 0.012  EF_fuel CO2_=0.003  EF_fuel CH4_=7.7x10^-5^  EF_fuel N2O_ =2.2x10^-6^  EF_elec CO2_=6.44x10^-5^  EF_elec CH4_=1.6x10^-6^  EF_elec N2O_ =4x10^-7^ | 3.x10^-7^  EF_D AD CO2_ =0.44  EF_fuel CO2_=0.003  EF_fuel CH4_=7.7x10^-5^  EF_fuel N2O_ =2.2x10^-6^  EF_elec CO2_=6.44x10^-5^  EF_elec CH4_=1.6x10^-6^  EF_elec N2O_ =4x10^-7^  EF_ID AD_=-0.1 | 0.573  EF_D I F CO2_=0.79  EF_ID I_=-0.05 | 0.832  EF_Lf F CH4_=2.063  EF_fuel CO2_=0.003  EF_fuel CH4_=7.7x10^-5^  EF_fuel N2O_ =2.2x10^-6^  EF_elec CO2_=6.44x10^-5^  EF_elec CH4_=1.6x10^-6^  EF_elec N2O_ =4x10^-7^ |
| WARM | Aggregated  Disaggregated |  | -0.184  EF_fuel CO2_ = 0.003  EF_Co F CH4_= 0.00462  EF_Co F N2O_=0.041  EF_Co F CS_=-0.24 |  | -0.12  EF_I F N2O_=0.04  EF_ID I F_=-0.16 | 0.578  EF_fuel F CO2_=0.003  EF_Lf F CH4_=1.63  EF_Lf F CS_=-0.08 |
| *Paper* |  |  |  |  |  |  |
| IPCC-2006 | Aggregated  Disaggregated |  |  |  | 0.034  EF_D I P CO2_=0.015  EF_D I P N2O_=0.017  EF_D I P CH4_=0.001 | 1.590  EF_Lf P CH4_ =3.975 |
| EpE |  |  |  |  |  |  |
| IWM | Aggregated  Disaggregated | -0.83 |  |  | -1.1  EF_D I N2O_ =0.33  EF_D I P_=-1.43 | 0.684  EF_Lf P CH4_ =1.68  EF_Lf N2O_ =0.013  EF_fuel CO2_=0.0027  EF_elec CO2_=1.14x10^-3^  EF_elec CH4_=0.02x10^-3^  EF_elec N2O_ =0.6x10^-3^ |
| IWM-2 | Aggregated  Disaggregated |  | 0.012  EF_fuel CO2_=0.003  EF_fuel CH4_=7.7x10^-5^  EF_fuel N2O_ =2.2x10^-6^  EF_elec CO2_=6.44x10^-5^  EF_elec CH4_=1.6x10^-6^  EF_elec N2O_ =4x10^-7^ | 3.x10^-7^ EF_D AD CO2_ =0.44  EF_fuel CO2_=0.003  EF_fuel CH4_=7.7x10^-5^  EF_fuel N2O_ =2.2x10^-6^  EF_elec CO2_=6.44x10^-5^  EF_elec CH4_=1.6x10^-6^  EF_elec N2O_ =4x10^-7^  EF_ID AD_=-0.1 | 1.24  EF_D I P CO2_=1.28  EF_ID I_=-0.05 | 0.832  EF_Lf P CH4_= 2.063  EF_fuel CO2_=0.003  EF_fuel CH4_=7.7x10^-5^  EF_fuel N2O_=2.2x10^-6^  EF_elec CO2_=6.44x10^-5^  EF_elecCH4_=1.6x10^-6^  EF_elec N2O_ =4x10^-7^ |
| WARM | Aggregated  Disaggregated | -3.520 |  |  | -0.42  EF_I P CO2_ =0.03  EF_ID I P_= -0.45 | 0.036  EF_fuel CO2_ =0.003  EF_Lf P CH4_=2.1  EF_Lf P CS_=-0.81 |
| *Plastics* |  |  |  |  |  |  |
| IPCC-2006 | Aggregated  Disaggregated |  |  |  | 2.219  EF _D I PL CO2_ =2.2  EF_D I PL N2O_=0.017  EF _D I PL CH4_ =0.001 | 0 |
| EpE |  |  |  |  |  |  |
| IWM | Aggregated  Disaggregated | -4.530 |  |  | -1.71  EF _D I N2O_=0.33  EF _D I CO2_ =0.98  EF _ID I Pl_= -3.02 | 0.020  EF_fuelCO2_=0.0027  EF_elecCO2_=1.14x10^-3^  EF_elecCH4_ =0.02x10^-3^  EF_elecN2O_= 0.6x10^-3^ |
| IWM-2 | Aggregated  Disaggregated | -1.203 |  |  | 2.652  EF _D I PL CO2_ =2.74  EF _ID I_ = -0.05 |  |
| WARM | Aggregated  Disaggregated | -0.980 |  |  | 1.56  EF _I PL CO2_ =2.4  EF _ID I PL_=- 0.84 | 0.006  EF _fuel CO2_ =0.003  EF _Lf PL CH4_=0  EF _Lf PL CS_=0 |
| *Textiles* |  |  |  |  |  |  |
| IPCC-2006 | Aggregated  Disaggregated |  |  |  | 0.253  EF_D I T CO2_ =0.235  EF_D I T N2O_=0.017  EF_D I T CH4_ =0.001 | 0.954  EF_Lf T CH4_ =2.385 |
| EpE |  |  |  |  |  |  |
| IWM |  |  |  |  |  |  |
| IWM-2 | Aggregated  Disaggregated | -5.869 |  |  | 1.24  EF_D I TCO2_ =1.28  EF_ID I_ = -0.05 | 0.832  EF_Lf T CH4_= 2.063  EF_fuel CO2_=0.003  EF_fuel CH4=_7.7x10^-5^  EF_fuel N2O_=2.2x10^-6^  EF_elec CO2_=6.44x10^-5^  EF_elec CH4_=1.6x10^-6^  EF_elec N2O_=4x10^-7^ |
| WARM | Aggregated  Disaggregated | -2.370 |  |  | 1.23  EF_D I T CO2_ =1.67  EF_ID I T_= -0.44 | 0.006  EF_fuel CO2_ =0.003  EF_Lf T CH4_=0  EF_Lf T CS_=0 |
| *Garden* |  |  |  |  |  |  |
| IPCC-2006 |  |  |  |  |  | 0.663  EF_Lf GA CH4_ =1.657 |
| EpE |  |  |  |  |  |  |
| IWM |  |  | 0.066 |  |  |  |
| IWM-2 |  |  |  |  |  |  |
| WARM | Aggregated  Disaggregated |  | -0.155  EF_fuel CO2_ =0.003  EF_Co GA N2O_ =0.06  EF_Co GA CS_=-0.24 |  | -0.19 EF_ID I GA_= -0.19 | 0.988  EF _fuel CO2_ =0.003  EF_Lf GA CH4_=0.88  EF_Lf GA CS_=0.63 |
| *Wood* |  |  |  |  |  |  |
| IPCC-2006 |  |  |  |  |  | 2.016  EF_Lf W CH4_ =5.04 |
| EpE |  |  |  |  |  |  |
| IWM |  |  |  |  |  |  |
| IWM-2 |  |  |  |  |  |  |
| WARM | Aggregated  Disaggregated | -2.460 |  |  | -0.4  EF _D I W N2O_=0.04  EF _ID I W_= -0.44 | -0.614  EF _fuel CO2_ =0.003  EF_Lf W CH4_=1.3  EF_Lf W CS_=-1.14 |
| *Glass* |  |  |  |  |  |  |
| IPCC-2006 |  |  |  |  |  |  |
| EpE |  |  |  |  |  |  |
| IWM | Aggregated  Disaggregated | -0.92 |  |  | 0.376  EF _D I N2O_=0.98  EF _D I CO2_ =0.34  EF _ID I G_= 0.05 | 0.020  EF_fuelCO2_=0.0027  EF_elecCO2_=1.14x10^-3^  EF_elecCH4_ =0.02x10^-3^  EF_elecN2O_= 0.6x10^-3^ |
| IWM-2 | Aggregated  Disaggregated | -0.087 |  |  | 0.094  EF _D I G CO2_ =0.059  EF _ID I_ = -0.05 |  |
| WARM | Aggregated  Disaggregated | -0.280 |  |  | 0.025  EF _ID I G_=0.025 | 0.006  EF_fuel CO2_ =0.003  EF_Lf G CH4_=0  EF_Lf G CS_=0 |
| *Metals* |  |  |  |  |  |  |
| IPCC-2006 |  |  |  |  |  |  |
| EpE |  |  |  |  |  |  |
| IWM | Aggregated  Disaggregated | -1.994 |  |  | 0.5  EF _D I N2O_=0.33  EF _ID I M_= 0.17 | 0.020  EF_fuel CO2_=0.0027  EF_elec CO2_=1.14x10^-3^  EF_elec CH4_ =0.02x10^-3^  EF_elec N2O_= 0.6x10^-3^ |
| IWM-2 |  | -4.553 |  |  |  |  |
| WARM | Aggregated  Disaggregated | -3.970 |  |  | -0.02  EF _ID I M_=-0.02 | 0.006  EF_fuel CO2_ =0.003  EF_Lf M CH4_=0  EF_Lf M CS_=0 |
| *Nappies* |  |  |  |  |  |  |
| IPCC-2006 | Aggregated  Disaggregated |  |  |  |  | 1.013  EF_Lf N CH4_ =2.532 |
| EpE |  |  |  |  |  |  |
| IWM |  |  |  |  |  |  |
| IWM-2 |  |  |  |  |  |  |
| WARM |  |  |  |  |  |  |
| *Others or commingled MSW* | |  |  |  |  |  |
| IPCC-2006 | Aggregated  Disaggregated |  | 0.177^(b)^  EF_Co CH4_= 0.084  EF_Co N2O_= 0.093 | 0.021  EF _AD CH4_= 0.021 | 0.022  EF _D I O CO2_ =0.003  EF_D I O N2O_=0.017  EF _D I O CH4_ =0.001 |  |
| EpE^(c)^ | Aggregated  Disaggregated |  | 0.175^(d)^  EF_Co CH4_= 0.107  EF_Co N2O_= 0.065  EF_fuel CO2_=0.0026  EF_elec CO2_=5x10^-4^ | 0.045^(e)^  EF_AD CH4_ =0.009  E_fuel CO2_=0.0026  EF_elec CO2_=5x10^-4^ | 0.382^(f)^  EF _I CO2_= 0.332  EF _I N2O_= 0.01  EF_fuel CO2_=0.0026  EF_elec CO2_=5x10^-4^ | 0.009^(g)^  EF_fuel CO2_=0.0026  EF_elec CO2_=5x10^-4^ |
| IWM | Aggregated  Disaggregated |  |  |  | -0.58  EF _D I N2O_=0.33  EF _ID I O_= -0.91 | 0.020  EF_fuel CO2_=0.0027  EF_elec CO2_=1.14x10^-3^  EF_elec CH4_ =0.02x10^-3^  EF_elec N2O_= 0.6x10^-4^ |
| IWM-2 | Aggregated  Disaggregated |  |  | 0.345^(h)^ | 1.24  EF _D I O CO2_ =1.28  EF _ID I_ = -0.05 |  |
| WARM | Aggregated  Disaggregated |  |  |  | -0.01  EF _D I O_=0.38  EF _ID I O_= -0.35 | 1.242  EF _fuel CO2_ =0.003  EF_Lf O CH4_=3.64  EF_Lf O CS_=-0.22 |

^(a)^ Methods:

- **Entreprises pour l’Environnemnent (EpE):** It accounts for gross and net direct emissions, as well as indirect emissions (e.g. electricity consumption) and avoided emissions from the recovery of energy and material. In order to calculate direct emissions from waste degradation in landfills, the user selects a common method and refers to the regulatory methodologies recommended by the authorities of the country where the site(s) is (are) located.
- **Integrated Waste Management Model for municipalities IWM:** accepted by Environment Canada to evaluate the environmental performance of various elements of an ISWM system [1,2].
- **The Integrated Waste Management Model-2 (IWM-2)** developed by McDougall et al. (2001) [3] is based on ISWM and is to the International Standards ISO 14040 series on LCA.
- **The U.S. Environmental Protection Agency (EPA) Waste Reduction Model (WARM):** is used to estimate emissions reductions in climate change impact assessment [4]. In its last release, WARM (v. 14) included 54 materials, products and mixed categories [5].

^(b)^ Considers total mass of MSW treated.

^(c)^ EpE considers for recycling only: Direct emissions from permanent combustion facilities and on-site mobile equipment; direct emissions from refrigerant / fluorinated gases released because of WEEE leakages / dismantling process and indirect emissions from electricity or purchased heat consumption [6].

^(d)^ Considers CH_4_ emissions from Organic fraction of MSW and N_2_O emissions from MSW.

^(e)^ Considers VFG (vegetable, fruit and garden wastes) continuous process, as well as emissions from fuel and electricity consumption, however, it does not provide a methodology to account for avoided emissions from energy recovery.

^(f)^ Considers direct CO_2_ and N_2_O emissions from waste combustion as well as emissions from fuel and electricity consumption, however, it does not provide a methodology to account for avoided emissions from energy recovery.

^(g)^ To calculate emissions from landfills the entity should refer to the regulatory methodologies recommended by the competent authorities of the country where the site(s) is (are) located. It also considers direct emissions from (Direct emissions from permanent combustion facilities and on-site mobile equipment) and indirect emissions from (Indirect emissions from electricity or purchased heat consumption) and avoided emissions from electricity and heat recovery.

^(h)^ Organic MSW using bio gasification with electricity generation also considers biological treatment (composting or bio gasification) of paper waste.

**Disaggregated EFs** are by definition generic factors determined from a number of processes representing characteristics calculated per unit of activity; thus they are expressed in MTCO_2_E per characteristic unit (tonne of MSW treated; KWh of electricity; Liter of Diesel fuel) using a GWP_100_, (IPCC, 1995) [7]. EFs are fixed default values within every method with the exception of EpE where the user can select EFs of recycling (adapted from- [4] and landfilling (selected FOD method adapted from IPCC-2006 Guidelines [8].

**Aggregated EFs** is the combined impact of disaggregated EFs expressed in MTCO_2_E per tonne of waste category whereby LFG (landfill gas collected) = 0.6; Electricity consumed= 32 kWh/tonne of waste composted, 70-80 kWh/tonne of waste incinerated, 68-50 kWh/tonne of waste anaerobically digested, and 8kWh/ tonne of waste landfilled; Fuel consumed = ~2 Liters/ tonne of waste landfilled, ~3.28 Liters/ tonne of waste composted, and 0.89 Liters/ tonne of waste anaerobically digested.
**Co**= Composting; **I**=Incineration; **Lf**=Landfilling; **D**= Direct, **ID**= Indirect; **CS**= Carbon storage; **EFfuel**=Emission factor for fuel combustion; **EFelec**=Emission factor of electricity consumed or recovered; **CS**= Carbon storage; **CO_2_=**Carbon dioxide; **CH_4_**=Methane; **NO_2_**=Nitrous oxide; **F** = Food waste; **G** = Glass; **M** = Metals; **O** = others; **P** = Paper; **PL** = Plastics; **T** = Textiles; **W** = Wood; **GA**=Garden waste; **N**=Nappies.

**References**

[1] A. K. Mohareb, M. A. Warith, R. Diaz, Modelling greenhouse gas emissions for municipal solid waste management strategies in Ottawa, Ontario, Canada, Resour. Conserv. Recy. (2008) 1241-1251.

[2] EPIC and CSR (Environment and Plastics Industry Council and Corporations Supporting Recycling), Integrated waste management model for municipalities ON: Microsoft Excel Model, University of Waterloo, Waterloo. http://www.iwm-model.uwaterloo.ca/english.html, 2004 (accessed 03.08.14).

[3] F. McDougall, P. White, M. Franke, P. Hindle, Integrated Solid Waste Management: A Lifecycle Inventory, second ed., Oxford, 2001.

[4] EPA/ICF, Documentation for Greenhouse Gas Emission and Energy Factors Used in the Waste Reduction Model (WARM): Background Chapters (WARM V. 12), U.S. Environmental Protection Agency Office of Resource Conservation and Recovery, Washington DC, 2012.

[5] EPA/ICF, Documentation for Greenhouse Gas Emission and Energy Factors Used in the Waste Reduction Model (WARM): Background Chapters, U.S. Environmental Protection Agency Office of Resource Conservation and Recovery, Washington DC, 2016

[6] EpE, Protocol for the quantification of GHG emissions from waste management activities, Cedex, Nanterre, 2013.

[7] IPCC, B.8 Global Warming Potential (GWP): in IPCC Second Assessment Report (SAR) - Climate Change 1995. <http://www.ipcc.ch/ipccreports/sar/wg_I/ipcc_sar_wg_I_full_report.pdf>, 1995 (accessed 02.01.13).

[8] IPCC, Guidelines for National Greenhouse Gas Inventories, in: H.S. Eggleston, L. Buendia, K. Miwa, T. Ngara, K. Tanabe, (Eds.), National Greenhouse Gas Inventories Programme, IGES, Japan, 2006.
